# A Tale of Two Viruses: Immunological Insights Into HCV/HIV Coinfection

**DOI:** 10.3389/fimmu.2021.726419

**Published:** 2021-08-12

**Authors:** Samaa T. Gobran, Petronela Ancuta, Naglaa H. Shoukry

**Affiliations:** ^1^Centre de Recherche du Centre hospitalier de l’Université de Montréal (CRCHUM), Montréal, QC, Canada; ^2^Département de microbiologie, infectiologie et immunologie, Faculté de Médecine, Université de Montréal, Montréal, QC, Canada; ^3^Department of Medical Microbiology and Immunology, Faculty of Medicine, Zagazig University, Zagazig, Egypt; ^4^Département de médecine, Faculté de médecine, Université de Montréal, Montréal, QC, Canada

**Keywords:** human immunodeficiency virus, hepatitis C, coinfection (HIV infection), direct acting antiviral, anti retro viral therapy, liver fibrosis, CD4 T cell

## Abstract

Nearly 2.3 million individuals worldwide are coinfected with human immunodeficiency virus (HIV) and hepatitis C virus (HCV). Odds of HCV infection are six times higher in people living with HIV (PLWH) compared to their HIV-negative counterparts, with the highest prevalence among people who inject drugs (PWID) and men who have sex with men (MSM). HIV coinfection has a detrimental impact on the natural history of HCV, including higher rates of HCV persistence following acute infection, higher viral loads, and accelerated progression of liver fibrosis and development of end-stage liver disease compared to HCV monoinfection. Similarly, it has been reported that HCV coinfection impacts HIV disease progression in PLWH receiving anti-retroviral therapies (ART) where HCV coinfection negatively affects the homeostasis of CD4^+^ T cell counts and facilitates HIV replication and viral reservoir persistence. While ART does not cure HIV, direct acting antivirals (DAA) can now achieve HCV cure in nearly 95% of coinfected individuals. However, little is known about how HCV cure and the subsequent resolution of liver inflammation influence systemic immune activation, immune reconstitution and the latent HIV reservoir. In this review, we will summarize the current knowledge regarding the pathogenesis of HIV/HCV coinfection, the effects of HCV coinfection on HIV disease progression in the context of ART, the impact of HIV on HCV-associated liver morbidity, and the consequences of DAA-mediated HCV cure on immune reconstitution and HIV reservoir persistence in coinfected patients.

## Introduction

Hepatitis C virus (HCV) and human immunodeficiency virus type 1 (HIV-1) are two chronic viral infections that affect millions worldwide. They share similar routes of transmission through percutaneous exposure to infected blood, sexual activity and vertical transmission from infected mother-to-child. HCV is transmitted 10-times more efficiently than HIV through percutaneous exposure resulting in a high rate of HCV/HIV coinfection among people who inject drugs (PWID) (1). HIV infection on the other hand increases the risk of HCV acquisition through sexual contact, as demonstrated by recent outbreaks of HCV among men who have sex with men (MSM) (2, 3). Similarly, HIV infection increases the risk of vertical transmission of HCV from a mother to her infant (4, 5). Finally, people living in resource-limited countries with reduced access to diagnosis and medical care are at higher risk of being infected with both viruses (6). In absence of an effective vaccine against neither HCV nor HIV, coinfection is a seriously growing public health problem, with 2.3 million individuals affected, of whom 1.3 million are PWID (7).

The immunopathology of HCV/HIV coinfection is more deleterious than each infection separately. HIV accelerates the development of HCV-related liver disease including advanced liver fibrosis, cirrhosis, hepatocellular carcinoma (HCC) and death (8). Similarly, HCV coinfection is a major cause of non-AIDS-related morbidity and mortality in people living with HIV (PLWH) as it is associated with reduced or slower CD4^+^ T cell reconstitution after antiretroviral therapy (ART) (9). In addition, recent studies have reported that direct acting antiviral (DAA)-mediated HCV cure leads to an increase in cell-associated HIV-DNA in the blood of ART-treated PLWH who had low viral reservoirs at baseline, likely as a consequence of viral reservoir mobilization from tissues (10, 11). Whether DAA-mediated cure of HCV represents an obstacle to HIV reservoir elimination in coinfected subjects remains an important question to address.

In this review, we will discuss the interplay between HIV and HCV during coinfection. Specifically, how HIV influences HCV infectious outcome and liver disease progression and how HCV coinfection modulates the HIV latent reservoir and immune reconstitution following DAA-mediated cure and relevance to clinical management of coinfected patients.

## HCV Monoinfection

### Epidemiology

HCV is transmitted primarily through exposure to contaminated blood. Sexual transmission and vertical transmission from an infected mother to her infant are low, but both transmissions increase in the context of HIV coinfection (3–5). It is estimated that 71 million individuals are chronically infected with HCV globally (12). Injection drug use is the main source of HCV infection in high income countries, while unsafe medical injections/procedures are the main source in the developing world (13). The opioid crisis in the USA, has led to doubling the national incidence of HCV infection between 2010 and 2014 and the numbers continue to rise (14). Globally, drug use accounts for ~23% of new HCV infections (15).

### Viral Replication Cycle

The HCV genome consists of an uncapped positive single stranded RNA of approximately 9.6 kb-pairs [reviewed in (16)]. The genome represents an uninterrupted open reading frame (17), encoding a polyprotein precursor of approximately 3,000 amino acids including three structural proteins (Core, envelope glycoproteins E1, and E2) and seven non-structural (NS) proteins (P7, NS2, NS3, NS4A, NS4B, NS5A, and NS5B) (16). HCV replicates primarily in human hepatocytes. Although HCV uses multiple receptors for entry into host cells, it is dependent on four main receptors on the surface of hepatocytes: CD81, a cell membrane tetraspanin protein, the scavenger receptor class B type I (SR-BI), and the tight junction proteins claudin-1 (CLDN1) and occludin (OCLN) (16, 18). The viral envelope glycoproteins fuse with the cellular membrane by clathrin-mediated endocytosis then the viral genome is released into the cytosol (16). This is followed by translation of the open reading frame of the HCV genome generating a large polyprotein that is later processed into mature structural and non-structural proteins (16). Junctions between structural proteins are processed by host signal peptidases while the non-structural proteins are processed by the NS2/3 autoprotease and the NS3/4A serine protease. Replication of the HCV RNA takes place within endoplasmic reticulum derived structures known as the membranous web through a negative strand intermediate in a replication complex (16). This is mediated by the viral NS5B protein that acts as an RNA-dependent RNA polymerase and regulated by the NS5A protein that plays many pleiotropic functions during HCV replication and assembly (19). The progeny virion is assembled into a nucleocapsid built from the structural core proteins around the viral RNA. This is followed by virion release with a surrounding membrane derived from the human cell with embedded heterodimers of the envelope glycoproteins E1 and E2 (16).

### Natural History, Pathogenesis, and Immune Responses

The acute phase of HCV is empirically defined as the first 6 months post infection. Acute HCV is asymptomatic in the majority of infected subjects. Approximately 20–30% of infected subjects are able to clear the virus spontaneously during the acute phase while 70–80% develop persistent infection (20). As the virus continues to replicate in hepatocytes, it elicits persistent inflammation with increased expression of pro-inflammatory cytokines and chemokines from hepatocytes, the liver resident macrophages known as Kupffer cells, and other immune cells such as monocyte derived macrophages, natural killer (NK) cells and dendritic cells (DCs) that are recruited to the liver. Virus replication in hepatocytes also triggers hepatocyte damage with production of reactive oxygen species (ROS), damage associated molecular patterns (DAMPs) and some apoptosis. Altogether these mediators trigger the fibrogenic process (21). Hepatic stellate cells (HSCs), the main mediators of fibrosis, are activated by these different inflammatory signals and the cytokine TGF-β and start to express alpha-smooth muscle actin (αSMA) and collagen type I. Persistent inflammation leads to continued deposition of matrix proteins and imbalance in their degradation through increased expression of tissue inhibitors of metalloproteases (TIMPs) leading to liver stiffness and gradual loss of function (22). This chronic liver damage can progress to different stages of fibrosis, cirrhosis and HCC over 5-30 years (20). Multiple host factors including age, male sex, alcohol consumption and/or coinfection with HIV can accelerate progression to end-stage liver disease (23, 24).

HCV infection of hepatocytes triggers innate immune responses that are reviewed extensively elsewhere (25). Briefly, the cytosolic RIG-I-like receptor (RLR), retinoic acid-inducible gene-I (RIG-I), recognizes 5′-triphosphate double-stranded RNA (dsRNA) replicative intermediate and/or polyuridine (poly(U)) motifs within the HCV RNA. RIG-I then translocates to the mitochondria where it interacts with the mitochondrial antiviral-signaling protein (MAVS) to activate the downstream transcription factors IRF-3 and NF-κB, resulting in the induction of type I and type III interferons (IFNs) (25). HCV dsRNA is also sensed by the toll-like receptor 3 (TLR3) resulting in innate immune signaling through the adaptor molecule TRIF. HCV is able to evade innate immune responses by virtue of its NS3/4A protease that cleaves MAVS and inactivates its downstream signaling effect(s) (25) and by blocking the TLR3-mediated interferon signaling *via* NS4B-induced TRIF degradation (26). Finally, infected hepatocytes also shed exosomes carrying HCV RNA that are taken up by liver infiltrating plasmacytoid DCs (pDCs) that are then activated to produce type I and type III IFNs in the liver microenvironment (27). This IFN response is reflected as increased levels of interferon-induced genes (ISG), chemokines and inflammatory mediators in the liver thus activating liver resident inflammatory cells (28, 29). This response is also detectable in the peripheral blood. One notable ISG is CXCL-10 or interferon-gamma-inducible protein-10 (IP-10) that is elevated in plasma during acute HCV where high levels can predict failure to spontaneously resolve HCV (30). Levels of ISG induction correlate with single nucleotide polymorphisms in the IFN-lambda 3 (IFNλ3)/IFNλ4 region that are also associated with acute infection outcome [reviewed in (31)]. Following spontaneous resolution, ISG and inflammatory mediators return to normal levels but remain elevated in those who develop chronic infection (32). Natural killer (NK) cells are also activated during acute infection irrespective of infectious outcome and exhibit reduced production of cytokines and enhanced cytotoxic functions in chronic infection [reviewed in (33)].

A broad, polyfunctional and sustained virus-specific CD4^+^ and CD8^+^ T cell response is essential for spontaneous viral clearance [reviewed in (34)] where CD127^+^ virus-specific memory T cells develop (35–38). The abrupt disappearance of HCV-specific CD4^+^ helper T cell responses compromises CD8^+^ T cell function(s) and facilitates emergence of viral escape mutants (39–42) resulting in chronic infection. In chronic HCV, CD8^+^ T cells become exhausted and express different levels of exhaustion markers like programmed death-1 (PD-1), T cell immunoglobulin and mucin domain-containing protein 3 (Tim-3), and others [reviewed in (34, 43)], and lose effector functions (34). This is associated with downregulation of the transcription factor T-bet and different levels of expression of the nuclear factors T cell factor 1 (TCF1), Eomesodermin (Eomes), and thymocyte selection-associated high mobility group box protein (TOX) that distinguish different subsets of exhausted CD8^+^ T cells (44–46). TCF1^+^CD127^+^PD1^+^T-bet^lo^ HCV-specific CD8^+^ T cells expressing both exhaustion and memory markers and of limited functionality were described in HCV chronically infected subjects and termed “memory-like” or “stem-like” T cells (47, 48). While PD-1^hi^Eomes^hi^TOX^hi^CD127^-^ were reported to be a terminally exhausted subset (49). Recent single cell RNA sequencing (scRNA-seq) analysis revealed that TCF1^+^CD127^+^PD1^+^ memory-like cells are likely the progenitors of the PD-1^hi^Eomes^hi^TOX^hi^CD127^-^ terminally exhausted cells (49). Finally, escape mutations that occur in epitopes targeted by CD8^+^ T cells influence their phenotype as they can no longer see their cognate antigen despite persistent viremia. CD8^+^ T cells targeting epitopes that have escaped, express fewer exhaustion markers and revert to a memory phenotype where they express the memory marker CD127 and acquire transcriptomic and functional signatures that partly resemble memory CD8^+^ T cells generated following spontaneous HCV clearance (50–52).

Increasing evidence suggest an important role for antibodies (Abs) against the HCV glycoproteins E1 and E2 in spontaneous clearance (53, 54). Development of neutralizing Abs (NAbs) is generally delayed during acute infection (55–57). Although Ab responses are short lived in resolvers (58), they appeared early during acute resolving infection(s) in some subjects following expansion of activated circulating T follicular helper cells (cTfh) expressing IL-21 that help expansion of HCV-specific B cells (59). Generation of NAbs correlated with spontaneous resolution in other studies (17, 60). Preincubation of virus inoculum with anti-HCV Abs or passive immunization resulted in reduced viral loads and/or sterilizing immunity in animal models (61–65). Broadly neutralizing Abs (bNAbs) neutralizing multiple HCV genotypes, blocked infection in humanized mice (66–68) and were isolated from spontaneous HCV resolvers (69) underscoring their protective role.

Spontaneous HCV clearance generates long-lived memory T cells with enhanced protective immunity upon reinfection (58, 70). Subsequent HCV reinfections are typically of lower viral loads, shorter viremia and higher clearance rate (80-50% *vs* 25%) (71–74). In contrast, correlates of long-term protective immunity upon repeated HCV exposures are not well understood. Depletion studies in chimpanzees underscored the essential and complementary protective roles of CD4^+^ and CD8^+^ memory T cells in preventing HCV persistence (42, 70). Protection in PWID was associated with increased magnitude, breadth and, polyfunctionality of HCV-specific memory T cells (71, 72) and NAbs (71). Altogether, these results suggest that long-term protective immunity against HCV is possible and effective and provide strong rationale for vaccine development.

### Treatments for HCV

For nearly 30 years, interferon-based therapeutic regimens were the only option for treatment of chronic hepatitis C. These regimens were long (lasting nearly a year), had numerous side-effects, and were successful in only 50% of the treated individuals. Direct acting antivirals (DAA), targeting several key steps in the HCV replication cycle, have become increasingly available in recent years (75). Currently approved DAA primarily target the NS3/4A protease, the NS5B polymerase and NS5A and are usually given in different combinations (76). DAA has substantially changed the management of chronic HCV, as HCV cure is achieved in >95% of cases (76). Nevertheless, DAA regimens are costly and access to diagnosis and treatment remains limited especially among the highest risk groups like PWID and MSM. Furthermore, DAA-mediated cure does not protect against HCV reinfection (77). Hence, the development of an effective prophylactic anti-HCV vaccine remains a priority to achieve HCV elimination.

## HIV Monoinfection

### Epidemiology

HIV-1 is transmitted by sexual contact across mucosal surfaces, by vertical transmission through maternal-infant exposure, and by percutaneous exposure (78). It is estimated that 37.9 million people are infected with HIV worldwide (79). HIV incidence continues to rise among PWID through sharing HIV-contaminated materials. In addition, unprotected sex is another important HIV risk factor among PWID, female sex workers, and MSM (80). HIV-infected PWID transmit HIV sexually to non-injectors, and through vertical transmission (81). Whereas overall, 10% of HIV-infected persons are coinfected with HCV, among PWID, HCV coinfection rates range from 50% to >90% (81). Other factors are associated with higher rates of HIV-1 acquisition such as high number of recent sexual partners, anal sex, concurrent sexually transmitted diseases, as well as the viral load and the clinical stage of the transmitting partner (82, 83). Access of PLWH to ART increased from 2.98 million in 2006 to 21.8 million in 2017 and was accompanied by a 51% reduction in HIV-associated mortality from 1.95 million in 2006 to 0.95 million in 2017. However, the annual incidence of new HIV infections decreased only by 17%. The combination of decreased mortality associated with ART implementation and only a small decrease in the incidence of new HIV infection has led to an overall important increase in the number of PLWH world-wide from 8.74 million in 1990 to 36.82 million in 2017 (84).

### Viral Replication Cycle

HIV belongs to the genus Lentivirus within the Retroviridae family, with a virion containing two copies of positive single stranded RNA. The HIV genome of 9.2 Kb contains 9 genes encoding for multiple structural (*gag*, *pol*, *env*), regulatory (*tat*, *rev*), and accessory (*nef*, *vpr*, *vif*, *vpu*) proteins. The *env* gene encodes for the envelope glycoprotein gp160, that is further processed into gp120 and gp41. gp120 mediates HIV entry into target cells (*e.g.* CD4^+^ T cells and macrophages) through interaction with the CD4 molecule, which is the main receptor for the virus (85), as well as the chemokine receptors CCR5 and/or CXCR4, which are the main HIV coreceptors (86, 87). The molecular tropism of HIV, dictated by the capacity of specific viral strains to use CCR5 and/or CXCR4 for entry led to classification of HIV into CCR5-tropic (R5), CXCR4-tropic (X4) or dual-tropic (R5/X4) strains (87). Once gp120 binds to CD4, the envelope undergoes conformational changes, exposing the chemokine receptor binding domains of gp120 and allowing its interaction with the coreceptors (88). This leads to further conformational changes allowing gp41 to expose its fusion peptide, which penetrates the cell membrane to deliver the viral capsid inside the target cells (89). The HIV reverse transcriptase (RT) encoded by the *pol* gene initiates the process of reverse transcription by copying the positive-sense single-stranded RNA genome into a complementary DNA (cDNA) (90). The cDNA and its complement form a double-stranded viral DNA, which is then integrated into the host cell’s genome by the viral integrase (IN) encoded by the *pol* gene as well (91, 92). HIV integration is followed either by active viral transcription and production of new virion progeny or viral latency, with integrated proviral DNA persisting in many cells and tissues as latent HIV reservoirs (90, 93). The persistence of latent reservoirs despite viral-suppressive ART represents a major barrier to HIV cure (94, 95).

### Natural History, Pathogenesis, and Immune Responses

The course of HIV infection includes three phases: acute, chronic, and Acquired Immunodeficiency Syndrome (AIDS) (96, 97). The acute HIV infection phase, between the time of HIV acquisition to seroconversion, can be further subdivided in four Fiebig stages (I-IV), based on the sequential detection of HIV RNA, proteins, and antibodies (98). The acute phase is associated with high levels of viremia and wide HIV dissemination into lymphoid organs (99), where the virus encounters its main targets: CD4^+^ T cells, macrophages and dendritic cells (100–102). The concurrent HIV-specific immunity initiated during the acute phase is associated with a dramatic decline in viremia (96, 97). However, this immunity is inadequate to suppress viral replication completely, with HIV replication persisting in lymph nodes even when plasma viremia is undetectable (96, 99). After the acute phase, most patients have a period of viral latency, in which the viral genome is stably integrated in the host cell genome (96, 99). While CD4 counts normalize in the peripheral blood at the end of the acute phase, alterations in CD4^+^ T cell homeostasis were documented in the gut-associated lymphoid tissue very early upon infection and persist during the chronic phase (96, 99, 103). These alterations are at the core of HIV pathogenesis, with a “leaky gut” being the source of microbial translocation, chronic immune activation, systemic inflammation and disease progression (104, 105). During the chronic phase, the state of viral latency is reversed upon activation of latently infected CD4^+^ T cells upon interaction with their cognate pathogens leading to productive infection (93) and massive depletion of central memory CD4^+^ T cells, which are the “self-renewing” source for tissue effector memory CD4^+^ T cells (97). The HIV-dependent depletion of CD4^+^ T cells is mediated through pyroptosis (106), apoptosis of uninfected bystander cells (107), and CD8^+^ cytotoxic T cell killing (108). When CD4^+^ T cell counts decline below the limit of 200 cells/μl of blood (109), cell-mediated immunity is severely compromised, resulting in AIDS, a fatal complication characterized by life-threatening opportunistic infections and cancers (97).

In the absence of ART, the duration of the chronic phase is highly variable and can last for several years. While the majority of PLWH progress rapidly to AIDS, a minority remain immune competent (long-term non-progressors, LTNP) and a subset of those efficiently control HIV replication (elite controllers, EC) (110). LTNP are often asymptomatic for 10–20 years with CD4^+^ T cell counts maintained within the normal range (>500 cells/μl) and plasma viral loads between 5,000 and 15,000 RNA copies/ml (111, 112). On the other hand, EC represent a small subset of the HIV-positive population (< 1%) who are able to control HIV viremia below the limit of detection in the absence of ART (113). Nevertheless, disease progression occurs as well in LTNP and EC, thus justifying the current recommendations of early ART initiation (114, 115). The natural control of HIV infection is achieved *via* multiple mechanisms including the expression of intrinsic restriction factors that limit HIV replication, the development of efficient cellular immune responses (116) in association with specific protective HLA class I alleles (117) and humoral immune responses. Furthermore, natural resistance to HIV acquisition was described in sex workers in Africa and represents a reference for studying correlates of HIV mucosal immunity (118). Finally, early treatment initiation renders a fraction of PLWH able to control HIV infection following treatment interruption (119), thus supporting the concept of “functional HIV cure” (120). However, about 10 to 40% of ART-treated individuals are immunological non-responders (INRs) (121, 122); these PLWH fail to reconstitute their CD4^+^ T cell frequencies and functions despite controlled viremia under ART (123). This is due to the incapacity of ART to clear HIV reservoirs and diminish inflammation (124). This sub-group is at higher risk for developing AIDS and non-AIDs related morbidity and mortality (124–127).

HIV-specific CD8^+^ T cells are also key determinants of the course of natural HIV infection. Almost all HIV infected individuals mount a strong virus-specific CD8^+^ T cell response during the acute phase and the polyfunctionality of these CD8^+^ T cells is key to long-term control of viremia and limiting disease progression [reviewed in (128)]. Many of the evasion mechanisms employed by HCV are also used by HIV including escape mutations in targeted epitopes and CD8^+^ T cell exhaustion and are detailed elsewhere (128). High-dimensional functional and phenotypic analysis revealed phenotypic heterogeneity among HIV-specific CD8^+^ T cells as well as common exhaustion signatures and pathways that are shared not only among chronic viral infections like HCV, HIV and LCMV but also in cancer (49, 129). These include increased expression of Eomes and TOX as key features of exhausted T cells associated with disease severity in HIV infection while CD8^+^ T cells expressing lower levels of inhibitory receptors like PD-1 and higher levels of CD127, TCF1 and/or CXCR5 were associated with higher functionality, proliferation and better control of viral replication (129–131). Moreover, CXCR5^+^CD8^+^ T cells express less inhibitory receptors levels with higher cytotoxicity (132), which could contribute to improved control of viral reservoirs in lymph nodes particularly since lymphoid tissue resident memory CD8^+^ T cells were shown to express higher levels of in CXCR5, compared with blood memory CD8^+^ T cells (133) and that higher frequencies of follicular CXCR5^+^ CD8^+^ T cells correlated with lower rates of peripheral viremia (134).

### HIV Treatment

ART transformed HIV infection into a manageable chronic disease (78). Multiple antiviral drugs are now available that target different steps of the HIV replication cycle including, in order of discovery, reverse transcription, protease-mediated virion maturation, entry, fusion, and integration (78). However, ART does not eradicate HIV and viral rebound occurs rapidly upon treatment interruption due to the persistence of latent HIV reservoirs in long-lived memory CD4^+^ T cells (94). To date, HIV cure was achieved in only two people who received bone marrow transplantation to treat cancer using donor cells carrying the delta 32 mutation in the *ccr5* gene (CCR5δ32/δ32) (135), a mutation known to confer resistance to CCR5-tropic HIV infection (86).

ART is also important as a preventative measure. Early initiation of ART reduces sexual transmission of HIV-1 (136), particularly in genetically linked HIV-1 infections among serodiscordant couples (137). Pre-exposure prophylaxis (PrEP) using daily oral doses of the combined nucleoside reverse transcriptase inhibitors, tenofovir and emtricitabine, conferred higher protection against HIV than placebo in controlled trials (138–140). Furthermore, an injectable, long-acting integrase inhibitor, cabotegravir, is safe and well-tolerated and two large efficacy trials are underway in uninfected high-risk individuals (140, 141). In the past ten years, broadly neutralizing antibodies (bNAbs) targeting different HIV-1 envelope epitopes isolated from PLWH with slow disease progression were engineered (142, 143) and are currently being tested for both efficacy and safety as a potential alternative or adjuvant for ART (144).

## HCV/HIV Co-Infection

### Epidemiology

Approximately 6.2% of PLWH are also co-infected with HCV. The odds of HCV infection are six times higher in PLWH than in the HIV-negative population, with the highest prevalence among PWID and MSM, consistent with shared routes of transmission (7). Although parenteral transmission remains the principal route of HCV transmission in PWID (7), sexual transmission of HCV occurs among HIV-positive MSM (3, 145, 146).

### Natural History, Pathogenesis, and Immune Responses

Multiple factors shape the natural history of coinfection including genotype/subgroup of both viruses, host factors and socioeconomic factors including access to screening and treatment services (147). As reviewed above, virus-specific CD8^+^ T cell responses are essential for spontaneous resolution of acute HCV infection. The magnitude, breadth and polyfunctionality of the HCV-specific CD8^+^ T cells are key determinants of infectious outcome. These functions are dependent on CD4^+^ T cell help (34), and virus persistence during acute HCV is temporally associated with abrupt loss and/or dysfunction of virus-specific CD4^+^ helper T cells (148). As demonstrated in CD4^+^ T cell depletion studies in the chimpanzee model of HCV, loss of CD4^+^ T cells function was associated with decline in frequency of virus-specific CD8^+^ T cells and their cytokine production resulting in accumulation of escape mutations in the targeted CD8 epitopes with impairment of HCV-specific immune responses (42). CD4^+^ helper T cells are also essential for optimal B cell function and production of neutralizing antibodies. Specifically, antigen-specific B cell affinity maturation, isotype class switching and differentiation into antibody-secreting plasma cells in germinal centers are regulated by direct contact with CD4^+^ Tfh cells (59, 149).

Given the central role of CD4^+^ T cells in coordinating antiviral immune responses, it is not surprising that the immune response to HCV in the context of an HCV/HIV coinfection is compromised. HIV infection impairs the immune response to HCV, including in people who have cleared HCV infection (150). HIV-1-infected individuals with spontaneous control of HCV remain at significant risk for a second episode of HCV viremia (150). The breadth and magnitude of HCV-specific CD8^+^ T cells correlated with CD4^+^ T cell count (151). Similarly, coinfection and CD4^+^ T-cell counts <350/mm^3^ was associated with a global decline in the anti-HCV envelope antibody response, including binding antibody titers, NAb titers, and NAb breadth (152). These immunological factors are the likely cause of the observed lower rate of spontaneous clearance of acute HCV in HIV coinfected subjects (5-10%), as compared to HCV monoinfection (20-30%) (153), especially in patients with low CD4^+^ T cell counts (154, 155). Furthermore, the overall reduction in CD4^+^ T cells, microbial translocation, and systemic immune activation have deleterious effects on the liver with accelerated progression of liver fibrosis, early onset of cirrhosis and HCC, as compared to monoinfection (8)(discussed in the next section).

## Liver Disease in HCV/HIV Coinfected Individuals

**Figure 1:**
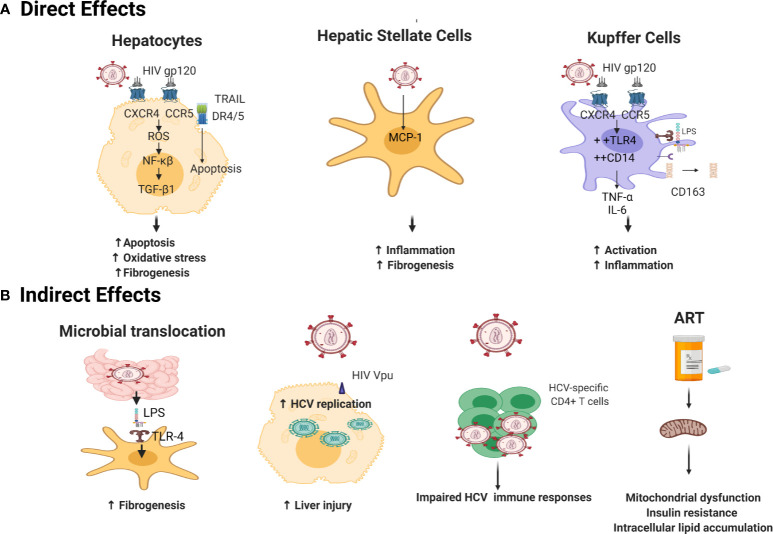
Mechanisms underlying acceleration of liver disease progression by HIV in HCV/HIV coinfection. **(A)** Direct Mechanisms: gp120 binds to HIV coreceptors (CXCR4 and CCR5) on hepatocytes surface (157) leading to accumulation of ROS which triggers an NF-κβ mediated oxidative stress (158, 159). HIV also activates TNF–related apoptosis-inducing ligand (TRAIL)-mediated apoptosis *via* upregulation of TRAIL receptor 1 (DR4), and 2 (DR5) (160), aggravating HCV fibrotic complications (161). HIV promotes hepatic stellate cells (HSC) collagen I expression and secretion of the proinflammatory cytokine monocyte chemoattractant protein-1 (MCP-1) inducing inflammation and fibrogenesis (162). HIV sensitizes Kupffer cells to lipopolysaccharide (LPS) *via* gp120 binding to CXCR4 and CCR5 (163) increasing cell surface expression of CD14 and TLR4, resulting in increased secretion of TNF-α and IL-6 (164). This is accompanied by shedding of CD163 in serum (165). **(B)** Indirect Mechanisms: HIV indirectly augments fibrosis mainly by microbial translocation: LPS translocate through impaired gut epithelium (105) and binds to Toll-like receptor 4 TLR4 on HSCs, leading to upregulated chemokine secretion and Kupffer cells chemotaxis (166). HIV accessory proteins Vpr (167) and Nef (168) enhance HCV RNA replication creating a state of inflammation and tissue damage. HIV causes functional alterations of HCV-specific immune responses with more HCV replication and hepatic inflammation (150, 169). ART elicits insulin resistance by altering mitochondrial function (170, 171) and increased intracellular lipid accumulation (172, 173) leading to enhanced development of liver fibrosis (174). Created with Biorender.com.

HCV/HIV co-infected individuals are at a higher risk of progressive liver disease, with accelerated progression to liver cirrhosis and higher rates of HCC, even when HIV is controlled by ART (24, 155, 156). HIV coinfection accelerates HCV-mediated fibrosis through both direct and indirect mechanisms that will be discussed in this section and are summarized in **Figure 1**.

### Direct Effect of HIV on Liver Disease Progression

HIV gp120 interacts with CXCR4 and CCR5 on the surface of hepatocytes and HSCs, respectively (157, 175). This interaction activates HSCs and promotes ROS accumulation through an NF-κB-dependent mechanism, resulting in enhancement of the fibrogenic process (158, 159). Co-culture experiments in the presence of HCV, HIV or both HIV and HCV demonstrated an additive effect of HIV on the profibrogenic program in hepatocyte and HSC lines through ROS, NF-κB, and TGF-β1 up-regulation; resulting in increased expression of the extracellular matrix protein collagen I, and TIMP1 (176).

It was also reported that HIV can infect HSCs in a CD4/chemokine coreceptor-independent manner. HIV infection promoted HSC collagen I expression and secretion of the proinflammatory chemokine monocyte chemoattractant protein-1 (MCP-1) (162). HIV was also shown to infect Kupffer cells, the liver resident macrophages that orchestrates the intrahepatic inflammatory response. Kupffer cells express the HIV receptor CD4 and both coreceptors CCR5 and CXCR4 [reviewed in (163)]. In vitro infection of Kupffer cells by HIV led to their sensitization to lipopolysaccharide (LPS) treatment by increasing cell surface expression of CD14 and TLR4, resulting in increased secretion of TNF-α and IL-6. This effect was maintained even after suppression of HIV replication using antiretroviral drugs (164). Activation of macrophages/Kupffer cells is mirrored by increased levels of the soluble macrophage activation marker CD163 in serum of HIV/HCV coinfected subjects accompanied by periportal CD163^+^ macrophage accumulation during fibrosis progression but not in established cirrhosis suggesting that soluble CD163 is probably a marker of active fibrogenesis rather than accumulated fibrosis (165).

HIV also enhances apoptosis of hepatocytes, another accelerator of the fibrogenic process. This effect is mediated by TNF–related apoptosis-inducing ligand (TRAIL) signaling and upregulation of TRAIL receptor 1 (DR4), and 2 (DR5) (160). The TRAIL-dependent hepatocyte apoptosis in HCV infected individuals is aggravated by HIV coinfection (161). In addition, HIV gp120 and HCV-E2 protein, can collaboratively elicit hepatocyte apoptosis *in vitro* through upregulation of Fas ligand expression and dephosphorylation of the anti-apoptotic molecule AKT (161, 177).

### Indirect Effect of HIV on Liver Disease Progression

The principal mechanism through which HIV indirectly accelerates liver disease progression is increased microbial translocation. HIV infection induces severe depletion of the gastrointestinal lymphoid tissue, impairing the gastrointenstinal tract epithelial integrity and allowing bacterial LPS to translocate from the gut to the systemic circulation and through the liver (105). LPS binds to TLR4 on the surface of quiescent HSCs, leading to upregulated chemokine secretion and chemotaxis of Kupffer cells. Concurrently, this interaction induces downregulation of the TGF-β pseudoreceptor Bambi to sensitize HSCs to TGF-β-induced signals from Kupffer cells (166). Activation of HSCs is further aggravated by HIV-mediated depletion of Kupffer cells that are responsible for LPS phagocytosis (163, 178).

Elevated levels of microbial translocation markers (e.g. LPS, LPS binding protein, soluble CD14) were reported in HCV-infected subjects upon HIV acquisition and compared to HIV uninfected subjects (179). In addition, decreased levels of endotoxin-core antibodies (EndoCAb IgM) and increased levels of IgG, specific for a heterophilic (alpha-galactose) epitope, which has previously been associated with stage of liver disease (180), were observed in HIV-infected compared to uninfected subjects (179). All these measures were strongly associated with HCV-related liver disease progression to cirrhosis (179).

Other indirect mechanisms include the enhancement of HCV RNA replication *via* the HIV accessory proteins Vpr (167) and Nef (168); the accumulation in the liver of HIV-specific CD8^+^ T cells creating a state of inflammation and tissue damage in co-infected patients (181, 182); and functional alterations of HCV-specific immune responses in the presence of HIV coinfection (150, 169). Also, the CD4/CD8 functional imbalance seen during HIV infection modifies the hepatic cytokine milieu, in favor of a profibrotic state in the liver (155, 183). Finally, HIV induces upregulation of the Triggering Receptor Expressed on Myeloid cells 1 (TREM1) on Kupffer cells and induces extracellular signal-regulated protein kinases 1 and 2 (ERK1/2) cascade leading to increased macrophage inflammatory response (184).

The effect of ART on liver injury in co-infected patients is controversial. Early studies suggested that nucleoside reverse transcriptase inhibitors (NRTIs) and protease inhibitors may affect mitochondrial function leading to increased insulin resistance (170, 171), and development of liver fibrosis (174). This effect is mainly mediated by polymerase-gamma suppression, a DNA polymerase necessary for mitochondrial DNA replication (185). Another proposed mechanism for ART-induced mitochondrial insufficiency is the inhibition of mitochondrial adenylate kinase and adenosine nucleotide translocator (186, 187). Studies using hepatocyte cell lines have demonstrated that non-nucleoside reverse transcriptase inhibitors (NNRTIs) can interfere with mitochondrial function leading to increase intracellular lipid accumulation which can further enhance liver damage (172, 173). In contrast, other reports suggested that ART enhances HCV-specific T cell responses with a significant decline in HCV-RNA levels (188) and reduction in the rate of hepatic decompensation events and liver fibrosis (189, 190). Additional studies are needed to evaluate the effect new regimens of ART on liver functions.

## Effect of HCV Infection on HIV Disease Progression

HCV infection impacts the natural history of HIV with increased risk of developing AIDS defining illness like HIV-related bacterial and mycotic infections in coinfected subjects (191). HCV likely contributes to increased immune activation in chronic HCV/HIV coinfection resulting in impaired immune responses and rapid progression to AIDS (192). The different effects of HCV on HIV disease progression are presented in this section and summarized in **Table 1**.

**Table 1:** Impact of HCV on HIV disease progression.

HCV-mediated immunological and virological alterations in PLWH	Proposed Mechanism	Effect/Reversal upon DAA treatment
Compromised restoration of CD4^+^ T cell frequency during ART	HCV induced hepatic inflammation and immune activation lead to altered T cell homeostasis (193)	Partial restoration of T cell compartment and memory profile (194)
Deterioration of CD8^+^ T cell function	Persistent liver inflammation contributes to generalized exhaustion of CD8^+^ T cells with upregulation of exhaustion molecules like PD-1, Tim-3 and CD39 on total and virus-specific CD8^+^ T cells (195–197)	Partial reversal of CD8^+^ T cell exhaustion (49, 198)
Augmentation of HIV replication.	NS3/4A: activates binding of AP-1 to LTR, facilitated by Vpu leading to increased HIV RNA reverse transcription into cDNA to be integrated in the host genome (199, 200).	Unknown
	Core protein: binds to TRAF2, TRAF5 and TRAF6, aided by Nef, initiating NFKB cascade ending in LTR stimulation (201).	Unknown
Higher HIV reservoirs size during ART	Immune activation: immune activated CD4^+^ T cells provide targets for seeding of HIV reservoir (202)	Partial reversal of hepatic inflammation (11)
	Impaired HIV-specific cell mediated immunity responsible for clearance of HIV infected cells with high frequency of T-regs and IL-10 (203)	Unknown
	Permissiveness of HCV specific CD4^+^ T cells to HIV infection (unproven)	Unknown
Increased risk of developing AIDS defining conditions	Increased immune activation in chronic HCV/HIV co-infection resulting in impaired immune responses (191, 192)	Unknown

### Impact of HCV on CD4^+^ and CD8^+^ T Cells

HCV seroconversion of HIV-infected subjects is associated with reduced CD4^+^ T cell counts during the first few years and their reduced recovery after ART (204, 205). This deficit could be attributed to the nature of the liver as a lymphoid organ essential for maintaining T cell homeostasis, where hepatic inflammation induced by HCV infection leads to altered T cell homeostasis (193). Similarly, persistent liver inflammation may contribute to enhanced immune activation and generalized exhaustion of CD8^+^ T cells with the upregulation of various exhaustion molecule like PD-1, Tim-3 and CD39 on total and virus-specific CD8^+^ T cells (195–197).

### Modulation of HIV Replication by HCV

HCV proteins may modulate HIV replication through multiple mechanisms [reviewed in (206)]. The HCV non-structural protein NS3/4A can activate HIV transcription through its LTR by enhancing DNA binding of the transcription factor AP-1 (199). The NS3/4A effect on HIV transcription was facilitated by the HIV protein Vpu, which mediates ubiquitination and subsequent degradation of NS4A allowing the destabilization of the NS3/4A complex and the nuclear translocation of NS3 (200). Other studies demonstrated that both, HCV core protein and HIV Nef can bind directly to the tumor necrosis factor receptor-associated factor (TRAF) 2, TRAF5, and TRAF6 and initiate the NF-κB signaling cascade thus enhancing HIV replication in monocyte-derived macrophages (201). Another *in vitro* study demonstrated that HCV core potentiates HIV‐1 replication in macrophages *via* upregulation of TNF‐α and IL‐6 *via* TLR2, JNK, and MEK1/2‐dependent pathways. Furthermore, TNF‐α and IL‐6 secreted from HCV core‐treated macrophages reactivated pro-viral HIV-1 DNA in the latently infected monocytic U1 cell lines (207). In contrast, another study, using the HepG2 hepatocyte cell line, demonstrated that Tat-induced LTR activation was suppressed by HCV core protein restricting HIV-1 transcription and replication. This core mediated LTR inhibition was unchanged when NS3/4A protein was added to the culture. However, LTR activation and gene transcription were enhanced by infectious HCV virions suggesting the involvement of other viral or cellular proteins in this process (208).

### HIV Reservoirs in HCV Coinfected Subjects

Few studies to date explored the effect of HCV coinfection on the HIV reservoir size (7). A recent study has demonstrated a larger HIV reservoir size in resting CD4^+^ T cells in ART-treated HCV/HIV coinfected individuals with chronic HCV or who have spontaneously resolved HCV as compared to HIV mono-infected subjects (209). This increased HIV reservoir size represents a major obstacle to HIV elimination in those co-infected subjects and can be explained by several theories. First, increased immune activation during HCV coinfection, where persistently activated HCV-specific and non-specific CD4^+^ T-cells provide new targets for HIV infection and latency, as previously observed with herpesviruses (202). This T cell activation may facilitate *de novo* infection, especially since the concentrations of antiretroviral drugs reaching lymphoid tissues are low (210). Another theory is the possibility that impaired HIV-specific cell-mediated immunity, responsible for clearance of HIV-infected cells, contributes to HIV reservoir persistence. This immune suppression may be a consequence of chronic HCV coinfection where higher frequencies of peripheral regulatory T cells (Tregs) and IL-10 producing cells were detected as compared to HIV monoinfected subjects (203). Finally, whether HCV-specific CD4^+^ T cells represent HIV infection targets and sites of HIV reservoir persistence remains to be investigated. Previous studies reveled that in addition to HIV-specific CD4^+^ T cells (211), increased HIV infection occurs in mycobacterium tuberculosis (MTb)-specific CD4^+^ T cells, while cytomegalovirus (CMV)-specific CD4^+^ T cells showed impaired permissiveness to HIV (212). Recently, several groups characterized the antigenic specificity of CD4^+^ T cells carrying HIV reservoirs, in an effort to therapeutically prevent HIV reservoir seeding in pathogen-specific cells or to target their specific elimination in HIV cure strategies (213, 214). We have recently demonstrated that integrative HIV infection in *Staphylococcus aureus*-reactive CD4^+^ T cells can be promoted by DCs in a retinoic acid-dependent manner (215) and the liver is an organ rich in retinoic acid (216). The liver is also an organ rich in Th7 cells (217), a subset of CD4^+^ T cells transcriptionally programmed to be HIV infection targets (218), with a considerable fraction of HCV-specific CD4^+^ T cells bearing the phenotypic and functional characteristics of Th17 cells (219). Therefore, it is tempting to speculate that HIV infection and viral reservoir persistence in HCV-specific CD4^+^ T cells may explain differences observed between HIV monoinfected and HIV/HCV coinfected individuals in terms of HIV reservoir size. The clonal expansion of HIV infected memory CD4^+^ T cells is a well-known mechanism, by which HIV latent reservoir is maintained and expanded (214, 220). A similar mechanism may be at play during concomitant HCV infection, potentially conferring preferential susceptibility of HCV-specific CD4^+^ T cells to HIV infection and reservoir expansion.

## Effect of DAA-Mediated Cure of HCV in Coinfected Subjects

Before the DAA era, HCV treatment and cure in HCV/HIV coinfected subjects was a complex problem since long IFN-based therapeutic regimens had multiple side effects and the rate of sustained viral response (SVR) was much lower than in monoinfected subjects. IFN-free DAA regimens that are widely available now have been a major game changer given their relative short treatment duration, reduced side effects and a response rate of >95%. In the context of HIV/HCV coinfection however, early studies suggested a lower SVR rate to DAA (86.3%) as compared to monoinfection (94.9%) (221). Nevertheless, most recent studies reported response rates to DAA in HIV/HCV coinfection as comparable to those in HCV-monoinfected individuals (222). Predictors of failure to achieve SVR under DAA in coinfected individuals are not different from those observed in monoinfected people and include sex, immune status, HCV RNA load, severity of liver disease, and the use of suboptimal DAA-based regimens (129). Given these promising results, HCV/HIV coinfected subjects are no longer considered difficult to reach an HCV cure. Nevertheless, specific consideration should be given to negative predictors of SVR and barriers to treatment that may be more common in the coinfected population (223). Data from several cohorts have identified a clear benefit of HCV cure in limiting liver disease progression and reducing the risk of developing HCC in PLWH, especially if they are treated at an early fibrosis stage (224, 225). In this section, we will discuss the impact of DAA-mediated cure of HCV on liver disease progression, immune reconstitution and the HIV reservoir in coinfected subjects. These points are also summarized in **Table 1**.

### Immune Reconstitution in HCV/HIV Coinfected Subjects Following DAA

DAA-mediated cure of HCV induces rapid decrease in serum levels of sCD163, a marker of inflammatory macrophage activity, and is associated with reduced histological inflammation in the liver (226). DAA therapy was also accompanied by a rapid decline in ISG expression in the liver and peripheral blood (227). Multianalyte profiling of 50 plasma proteins pre- and post DAA-mediated clearance in a cohort of 28 subjects with chronic HCV infection, including two HIV coinfected subjects, demonstrated that the elevated plasma cytokines and chemokines improve but do not completely normalize up to 8 months post cure (228). Interestingly, CXCL10/IP-10, an IFN induced chemokine, is usually elevated during chronic HCV infection and it rapidly decreased upon starting DAA therapy in line with reduced IFN-α signaling in the liver (228, 229). This was also associated with the normalization of NK cell phenotype and function (cytotoxicity and cytokine expression) (229)

The analysis of peripheral blood lymphocytes early during DAA therapy reported an increase in total CD4^+^ and CD8^+^ T cells but not NK cells or monocytes (230). At the same time, a reduction in activated (HLA-DR^+^ and CD38^+^) CD4^+^ and CD8^+^ T cells was observed in both monoinfected and coinfected subjects (230), with this effect being sustained up to one year post-SVR (231). Furthermore, there was an increase in the frequency of T cells expressing CXCR3, the CXCL10/IP-10 receptor (230), indicative of T cell redistribution in the periphery upon DAA treatment. Indeed, a reduction in liver inflammation may theoretically cause an efflux of lymphocytes from the liver and draining lymph nodes in the peripheral blood.

In HCV monoinfection, DAA treatment was associated with restoration of the proliferative capacity of HCV-specific CD8^+^ T cells (198). However, selective maintenance of TCF1^+^CD127^+^PD1^+^ memory like HCV-specific CD8^+^ T cells was observed following cessation of therapy (47), as well as a limited impact of DAA on the functional and mitochondrial impairment of HCV-specific CD8^+^ T cell responses (232). In contrast, Barili et al. reported partial improvement of the mitochondrial metabolic functions of HCV-specific CD8^+^ T cells following DAA treatment (233). However, they used *in vitro* stimulated PBMCs which may have affected mitochondrial function (233). Bulk RNA-seq and scRNA-seq analysis of HCV-specific CD8^+^ T cells identified an antigen-dependent core exhaustion signature, with memory-like CD8^+^ T cells targeting variant epitopes exhibiting a less pronounced exhaustion signature (49, 52). HCV-specific memory-like T cells harbored the same transcriptomic signatures before and after DAA treatment suggesting a permanent “exhaustion scar” that could not be reversed by DAA-mediated HCV cure (49). Only one study examined HCV-specific CD4^+^ T cells in subjects undergoing DAA treatment. In this study, the frequency of HCV-specific CD4^+^ T cells increased 2 weeks after starting DAA, with downregulation of the exhaustion and activation markers (CD38, CD39, ICOS, OX40 and PD-1) and upregulation of the memory-T cell markers (CD127 and CCR7) without complete normalization (234). As with total CD4^+^ T cells, this transient increase in HCV-specific CD4^+^ T cells early after starting DAA therapy is likely due to decreased liver inflammation and emigration of HCV-specific CD4^+^ T cells from the liver. It is noteworthy that HCV-specific CD4^+^ T cells with a Tfh phenotype and transcriptional signature were preferentially maintained following DAA-mediated HCV cure (234).

Conflicting results were reported in the setting of DAA therapy in HCV/HIV coinfection. One study reported a reduction in total activated (HLA-DR^+^ and CD38^+^) CD4^+^ and CD8^+^ T cells up to one year post-SVR (231). Another study did not observe any change in T cell activation at 12 weeks after DAA compared to baseline (235) but observed an increase in the frequency of total CD4^+^ and CD8^+^ T cells producing IFN-γ, IL-17, and IL-22 (235).

The frequency of Foxp3^+^CD25^+^CD4^+^ Tregs is usually elevated in chronic HCV infection and contribute to the immune suppressive and anti-inflammatory immune response in the liver. However, DAA-mediated clearance of HCV does not completely normalize frequency of Tregs in neither monoinfected (236) nor coinfected subjects (237). Similarly, the frequency of HLA-DR^-^CD33^+^ CD11b^+^ myeloid-derived suppressor cells (MDSC) are expanded in HCV monoinfected (238) and coinfected subjects (237). MDSC play a crucial role in immune suppression *via* production of arginase‐1, inducible NOS, TGF-β and IL-10 that inhibit T cell functions (239). The frequency of MDSC remains elevated post-DAA therapy in HCV/HIV co-infected subjects and may contribute to generalized immune suppression (237).

In summary, studies examining DAA-mediated cure of HCV, mostly in monoinfected subjects, suggest only partial reconstitution of immune functions and persistence of immune suppressive cells like Tregs and MDSC. This immune suppressive environment may contribute to reduced immune response to vaccinations, limited immune surveillance against cancer, and increased reactivation of latent or occult infections of herpesviruses and hepatitis B virus, respectively (240, 241). Finally, this incomplete reconstitution of HCV-specific CD4^+^ and CD8^+^ T cells may increase the risk of HCV persistence upon re-exposure and reinfection in high-risk populations like PWID and MSM. Additional studies examining immune reconstitution post DAA-mediated cure in HCV/HIV coinfected subjects are warranted.

### Liver Disease in HCV/HIV Coinfected Subjects Post DAA-Mediated Cure

As discussed above, DAA-mediated cure of HCV causes an improvement in liver fibrosis markers in both HCV monoinfected and HCV/HIV coinfected subjects (225, 242, 243). Furthermore, recent cohort studies suggest that HCV/HIV coinfected subjects with cirrhosis are no longer at higher risk for developing HCC or end-stage liver disease as compared to HCV monoinfection (225). However, the risk of developing HCC is not completely eliminated upon HCV cure and remains high in subjects with advanced fibrosis or cirrhosis pointing to an irreversible liver damage. Molecular studies using HCV-infected hepatocytes, as well as studies using liver biopsy samples from infected patients, have revealed that chronic HCV infection induces epigenetic and gene expression alterations associated with risk for HCC, alterations that persist after HCV cure (244, 245).

Other factors may contribute to continued risk of developing end-stage liver disease and HCC in HCV/HIV coinfected subjects. First, metabolic abnormalities including insulin resistance are quite common in HIV-infected individuals and result in hepatic steatosis with accumulation of triglycerides in hepatocytes, and can accelerate liver damage and increase risk of developing HCC irrespective of HCV status [reviewed in (246)]. Second, gut dysbiosis and intestinal damage are hallmarks of HIV infection that are not restored by ART [reviewed in (247)]. Considering the fact that DAA-mediated HCV clearance does not impact gut dysbiosis in cirrhotic HCV-monoinfected subjects (248), dysbiosis is highly likely to remain a prominent risk of liver damage in the context of HCV/HIV coinfection after DAA-mediated cure. Thus, new therapeutic interventions may be needed to normalize the metabolic status and restore intestinal health in DAA-treated HCV/HIV coinfected subjects.

### Impact of DAA Treatment on HIV Reservoir

The HIV reservoir levels have been intensively studied in ART-treated HIV infected subjects (249, 250), but little is known about HIV persistence in the context of HIV/HCV coinfection, especially following DAA treatment. A study performed on PBMCs revealed that DAA-mediated HCV clearance did not induce a decrease in HIV reservoirs but rather an increase in integrated HIV-DNA levels, mainly in patients with low versus undetectable levels of HIV viremia before DAA (251). Similarly, Rozera et al. observed a significant rise in HIV-DNA levels in the peripheral blood of some successfully ART-treated HIV/HCV co-infected patients at the end of DAA treatment, which correlated with lower cellular HIV reservoir at baseline (10). Most recently, a study of 97 ART-treated subjects reported that HCV/HIV coinfected subjects with chronic HCV infection, as well as HCV/HIV coinfected subjects who have spontaneously cleared HCV, showed higher levels of HIV-DNA in resting CD4^+^ T cells (CD25^-^CD69^-^HLADR^-^) compared to HIV monoinfected individuals (209). This increase in HIV reservoir size following DAA therapy may be explained by the mobilization of cells carrying HIV reservoirs from lymph nodes, liver and other tissues into the peripheral blood in response to DAA-mediated reduction of HCV-elicited immune activation and hepatic inflammation (10, 252). This is mirrored by the rapid decline of CXCL10 levels in response to DAA therapy, similar to plasma HCV RNA levels (253). In contrast, a new study demonstrated that although all three forms of HIV DNA (total, integrated, and episomal) remained stable during DAA treatment, cell-associated unspliced HIV-RNA levels (giving rise to the progeny virions) were significantly increased 12 months after the end of DAA therapy, suggesting an increased HIV transcriptional activity in reservoir cells (11). This may be explained by a decrease in IFN-mediated antiviral immunity as reflected by the observed reduction in ISG signals (227) and normalization of the expression levels of IFN-β, IFI44, and CXCL10 in the peripheral blood following DAA therapy (254). In addition, as reported by Meissner et al, liver biopsies from patients who achieved SVR showed that HCV cure was accompanied by decreased expression of type II and III IFNs, but higher expression of type I IFNs compared to pre-treatment baseline (227). Recently, diverse and opposing effects of the type I IFNα on HIV latency were described. IFNα inhibits the establishment of latency. However, once latency is established, IFNα is able to reverse it through binding to its receptor on CD4^+^ T cells and triggering phosphorylation of STAT1, 3, and 5 proteins (255). It is well-established that STAT5 can activate HIV transcription *via* its binding to the HIV long terminal repeat (256).

Altogether, the limited data available so far suggest that the HIV reservoirs are higher in HCV/HIV coinfected compared to HIV monoinfected subjects and that DAA-mediated HCV cure does not reduce HIV persistence. However, several limitations prevent the generalization of these findings because some studies used purified memory CD4^+^ T cells while others used total PBMCs to quantify HIV DNA (10, 11). Another issue is the extensive use of PCR-based techniques to assess the integrated HIV-DNA. These techniques tend to overestimate the size of the reservoir due to the high prevalence of defective proviruses (257). Other confounding factors include the pre-ART HIV viral load, host genetic factors and liver condition. Additional studies with well-defined cohorts and extended follow-up after DAA-mediated cure of HCV are essential to accurately evaluate the long-term effects of DAA therapy on the HIV reservoir.

## Conclusion

Since the discovery of HIV and HCV, tremendous advances were made in understanding the molecular steps of the viral replication cycle, natural infection, pathogenesis and immune responses. Studies also documented mechanisms by which HCV and HIV reciprocally influence their pathogenesis, thus leading to exacerbated alterations of immune competence as a consequence of impaired liver functions and altered intestinal barrier integrity. Despite the absence of efficient vaccines, DAA currently cures HCV infection, while ART controls viral replication at undetectable levels, thus improving the life quality of co-infected individuals. However, DAA treatment does not completely normalize immune and liver functions. Also, ART does not eradicate HIV reservoirs, which persist in long lived memory CD4^+^ T cells with various antigenic specificities, potentially including HCV-specific CD4^+^ T cells.

## Future Directions

New longitudinal studies should address the effect of DAA on HIV reservoir persistence in ART-treated HCV/HIV coinfected individuals in relation to age, sex, metabolic status, liver damage, drug and alcohol use, and other comorbidities. Given the fact that the pool of HCV-specific CD4^+^ T cells contracts but still persists upon DAA, studies are also needed to evaluate whether these cells contribute to the pool of latent HIV reservoirs in ART-treated individuals and whether HCV re-exposure and/or reinfection in high-risk groups may promote HIV reservoir expansion. Finally, additional therapeutic interventions may be needed to restore immune competence and control residual HIV transcription in ART-treated individuals after DAA-mediated HCV cure.

## Author Contributions

SG reviewed the literature and wrote the first draft. PA and NS reviewed the literature, revised the first draft, and modified the manuscript. All authors contributed to the article and approved the submitted version.

## Funding

Our research is funded through grants from the Canadian Institutes of Health Research CIHR (PJT-173467 (to NS), HOP-120239 and PJT-153052 (to PA)), the National Institutes of Health (NIH) (U01AI131313, R01AI136533 and U19AI159819 to NS), the Canadian HIV Cure Enterprise Team Grant (CanCURE 1.0) funded by CIHR in partnership with the Canadian Foundation for AIDS Research (CANFAR) and the International AIDS Society (IAS) (CanCURE 1.0; HIG-133050), and the CanCURE 2.0 Team Grant funded by CIHR (HB2-164064) to PA. SG is supported by a doctoral fellowship from the Canadian Network on Hepatitis C (CanHepC). CanHepC is funded by a joint initiative of the CIHR (NHC142832) and the Public Health Agency of Canada.

## Conflict of Interest

The authors declare that the research was conducted in the absence of any commercial or financial relationships that could be construed as a potential conflict of interest.

## Publisher’s Note

All claims expressed in this article are solely those of the authors and do not necessarily represent those of their affiliated organizations, or those of the publisher, the editors and the reviewers. Any product that may be evaluated in this article, or claim that may be made by its manufacturer, is not guaranteed or endorsed by the publisher.
